# Rare Case of Anti-LGI1 Limbic Encephalitis with New Onset Epilepsy: A Case Report

**DOI:** 10.7759/cureus.4608

**Published:** 2019-05-07

**Authors:** Mohan Kurukumbi, Jose A Castillo, Tulsi Shah, Rajesh Gupta

**Affiliations:** 1 Neurology, Inova Health System, Fairfax, USA; 2 Neurology, Virginia Commonwealth University School of Medicine, Richmond, USA

**Keywords:** limbic encephalitis, autoimmune encephalitis, encephalitis, anti-lgi1, seizure, epilepsy, rituximab, autoimmune brain disease, autoimmune disorders, brain mri

## Abstract

Anti-leucine-rich glioma-inactivated 1 (LGI1) limbic encephalitis (LE) has been classified as an autoimmune LE with a subacute course. Many patients with anti-LGI1 LE have normal or minimal cerebrospinal fluid (CSF) findings. Cerebrospinal fluid 14-3-3 protein or neuron specific enolase is usually seen in Creutzfeldt-Jakob disease (CJD) with high sensitivities, but can also be positive in other paraneoplastic and autoimmune encephalitides, which can make diagnosis challenging. The mainstay of treatment for anti-LGI1 LE generally focuses on steroids, intravenous immunoglobulin (IVIG), plasmapheresis, and/or rituximab. All the aforementioned modalities can be used in the treatment of anti-LGI1 LE and since this condition is highly responsive to treatment with steroids, prompt diagnosis can help stall the progression of this disease. Here, we present a case of anti-LGI1 LE that initially improved with empiric immunotherapy and showed definitive return to baseline with initiation of rituximab.

## Introduction

Anti-leucine-rich glioma-inactivated 1 (LGI1) limbic encephalitis (LE) is a subacute disorder characterized by faciobrachial dystonic seizures (FBDS), temporal lobe epilepsy, prominent short-term memory deficits, altered mental status (AMS) [1], and antibodies against the LGI1 subunit of the voltage-gated potassium channel (VGKC) complex on neurons [2]. These myoclonic findings typically precede or occur simultaneously with the other symptoms [1] and prompt testing for VGKC complex antibodies, which includes anti-LGI1. However, the workup for diagnosis is complex and treatment is not well standardized [3].

Here we report a case presenting with FBDS, progressive cognitive and memory decline, initially negative magnetic resonance imaging (MRI) and electroencephalogram (EEG) findings, and positive cerebrospinal fluid (CSF) protein 14-3-3 that dramatically improved with empiric treatment with immunotherapy as well as initiation of rituximab therapy. The patient was later confirmed to have positive anti-LGI1 antibodies in the CSF.

## Case presentation

A 72-year-old Caucasian female with a history of congenital right eye blindness, hypertension and anxiety was brought to the emergency department (ED) after a motor vehicle collision (MVC) secondary to her sudden onset of impaired awareness.

The patient had a sudden loss of awareness while driving, witnessed by her husband who was sitting in the passenger seat. She crossed several lanes and sideswiped and hit two cars before coming to a stop. The patient was totally amnestic regarding the event and was unaware of her course to the ED. History was negative for fecal and urinary incontinence, alcohol consumption, illicit drug use and recent head injuries. The family provided a recent three-month history of sporadic episodes of confusion that lasted for less than a minute each. During these episodes, the patient would suddenly become unaware of her surroundings, turn pale and stare into space or display inappropriate behaviors such as getting up from her seat during dinner and spitting her food into a vase. Resolution to baseline was spontaneous, but with no recollection of the event. The patient was intermittently alert and disoriented at the time of examination with flat affect, no spontaneous speech and unsteady gait. The rest of the neurological exam was normal.

Similar seizure occurrences were repeated during the ED and intensive care unit (ICU) stay and the patient received Keppra for seizure prophylaxis. Continuous EEG monitoring at this time was interpreted as background slowing compatible with a mild encephalopathic picture but no clear focal slowing or no electroclinical seizures.

Her cognitive decline was noted with a Montreal cognitive assessment (MOCA) score of 18, showing difficulties in areas of executive functioning, delayed recall, orientation, and abstraction. Brain magnetic resonance imaging (MRI) revealed no acute process. The patient was discharged on the 6th day of illness (DOI #6) after a negative workup with recommendations to do outpatient CSF studies and follow up with outpatient neurology. The patient was stable upon discharge.

On the 27th day of illness (DOI #27), the patient was readmitted to the hospital for continuing deteriorating mental status and increased frequency of staring episodes up to 28 times per day. The patient exhibited a baseline decline in cognitive function and personality changes. During this admission, her outpatient CSF workup results revealed an elevated white blood cell count at 9/mL with an elevated neutrophil and macrophage level at 34% and 4%, a high 14-3-3 protein level at 8.0 ng/mL and four well defined gamma restriction bands. At this time, her differential diagnosis was broadened with a concern for possible Creutzfeldt-Jakob disease (CJD) and herpes simplex encephalitis, and the patient was empirically treated with acyclovir. Repeat CSF studies done during this admission including an autoimmune profile, West Nile virus, herpes simplex virus 1 (HSV 1) and HSV 2, immunofixation electrophoresis, neuron specific enolase, rheumatoid factor (RF), antinuclear antibody (ANA) comprehensive panel, erythrocyte sedimentation rate (ESR), rapid plasma reagin (RPR) and venereal disease research laboratory (VDRL), Vitamin D, anti-neutrophil cytoplasmic antibody (ANCA) vasculitides antibodies, paraneoplastic profile of the serum and CSF all came back within normal limits.

**Figure 1:**
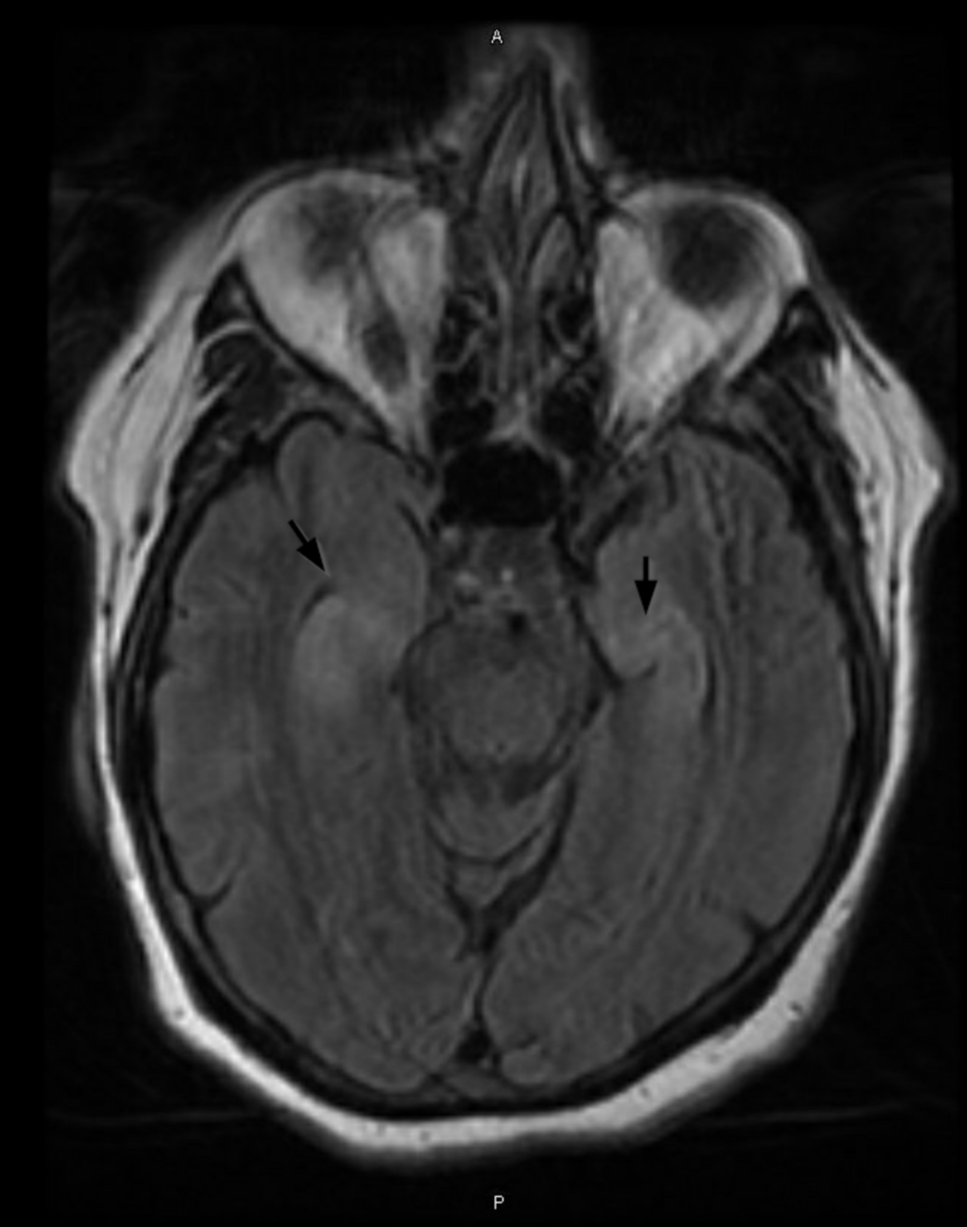
Relatively symmetric T2 prolongation and swelling of the bilateral hippocampal structures.

**Figure 2:**
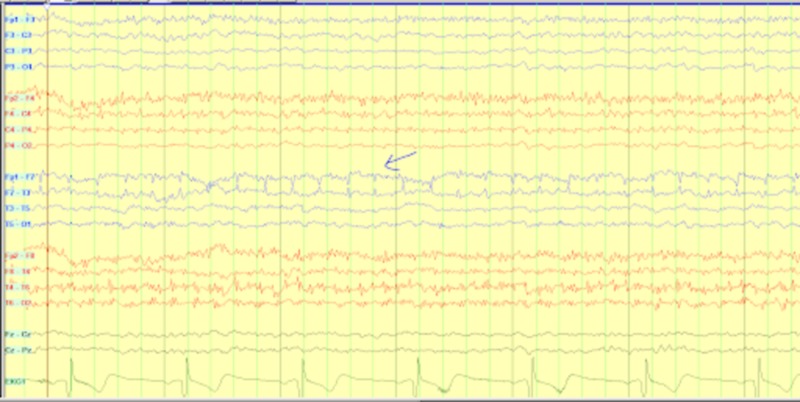
Continuous EEG showing left temporal discharges accentuated in frequency. EEG: Electroencephalogram

Interestingly, during this time the patient also had an increased appetite with fixation on sweet products, decreased short-term memory, ataxia, mild postural kinetic tremor bilaterally and exhibited obsessive behaviors such as continuously picking at her teeth and fingers. Keppra 500 mg twice a day was continued for seizure prophylaxis. A repeat brain MRI revealed symmetrical signal abnormality with swelling of hippocampi and right mesial temporal lobe, subtle T2 FLAIR signal abnormality, low-level restricted diffusion along the insular cortex, without associated contrast enhancement or hemorrhage, consistent with temporal pathology (Figure 1). The EEG record from previous hospitalization was re-evaluated by the attending neurophysiologist and revealed previously unseen focal discharge and seizure from the left temporal region (Figure 2). A repeat EEG during the second hospitalization was abnormal for slower background and generalized intermittent rhythmic slowing consistent with an encephalopathic picture, but the previously seen left temporal origin epileptiform discharges and electrographic seizures were not seen.

At this point, autoimmune LE and prion disease were still in the differential. Given her lack of improvement, it was deemed necessary to treat a reversible etiology like autoimmune LE vs. prion disease, the latter of which is irreversible. Pulsatile intravenous (IV) solumedrol, a high dose steroid, was started daily for five days. The patient showed marked improvements on steroids, with resolution of faciobrachial seizures on day 5 of this second admission (DOI #31) and improvement of mental function. She returned near baseline, with some passive lethargic behavior persisting.

Of note, during this hospitalization, the patient also had hyponatremia, initially 124 mEq/L that increased to 133 mEq/L after water restriction and 1 g of NaCl tablet. The patient was discharged on oral prednisone 60 mg and a H2 blocker for six weeks with plans for a repeat brain MRI after which a steroid taper would be started.

CSF started and performed by Mayo Clinic on DOI #21, returned positive on DOI #32 with antibodies specific for anti-LGI1 autoimmune LE.

The following year, at an outpatient visit with the neurology team, after she had been tapered off steroids, it was noted that she was having worsening of her memory concerning for regression, for which she was readmitted a third time. Repeat CSF at that time again showed anti-LGI1 antibodies, confirming relapse. At this time, she received five days of intravenous immunoglobulin (IVIG) and pulse steroids, which showed mild improvement, though she still exhibited mild expressive aphasia and recall difficulties. At this point, she was started on 1 gram of IV rituximab. At another outpatient follow-up, the patient and family endorsed dramatic improvement of cognitive function after the start of rituximab therapy and no continued AMS experiences.

## Discussion

Antibody-associated encephalitides are divided into antibodies directed against neuronal surface or intracellular antigens [2]. Anti-LGI1 LE belongs to the type with antibodies directed against the neuronal surface.

According to studies done previously, LGI1 encephalitis accounts for 11.2% of all autoimmune encephalitis cases. European studies have shown that the peak age of onset is between 61 and 64 years and that it is more prevalent in males, which accounted for about 55-66% of the patients [4].

LGI1 is a monogenic, human epilepsy-related neuronal protein, where mutations in LGI1 have been linked to familial autosomal dominant partial epilepsy [5]. The underlying mechanism behind this disorder is an antibody directed against LGI1.

So far, three molecular functions of LGI1 have been proposed: 1) LGI1 prevents inaction of Kv1 VGKC through KvB, 2) LGI1 regulates neuronal development of glutamatergic circuits in the hippocampus, and 3) LGI1 interacts with ADAM22/23 transmembrane proteins and regulates AMPA receptor-mediated synaptic transmission in the hippocampus [6].

Taking into account the third function of this protein, and that this protein is unique to neurons and functions as a ligand for proteins ADAM22 and ADAM23, it has been shown that these two proteins are associated with seizure activity when not bound to one another. This protein complex acts as a postsynaptic AMPA receptor scaffold to allow for the receival of a neurotransmitter in the hippocampus.

LGI1 antibodies were found to disrupt ligand-receptor interaction of LGI1 with ADAM22/23 complex which results in reversible reduction in synaptic AMPA receptors [7]. When lacking in LGI1, there is a reduction in AMPA receptor-mediated synaptic transmission and this has been shown in mice models to lead to seizures [6]. These proposed mechanisms help explain why our patient demonstrated hippocampal symptoms as well as seizures.

Anti-LGI1 LE has been classified as an autoimmune LE with a subacute course. One of the common manifestations seen in anti-LGI1 encephalitis has been shown to be seizures, memory disturbances, and cognitive deficits [1]. Disease course typically begins with simple partial seizures that are rapid but complex seizures may occur as seizures become more frequent [3]. The specific type of seizure this disorder typically presents with FBDS but these movements can easily be mistaken for dystonia or myoclonus.

FBDS is found to precede anti-LGI1 LE but it is not proven that FBDS inevitably precedes LE [1]. If present, FBDS typically occurs unilaterally but may also occur bilaterally, as was evident in our case. Several studies have also demonstrated that patients can frequently develop rapid eye movement, sleep behavior disorders and hyponatremia [8].

It has been shown that up to 60% of patients may have hyponatremia with anti-LGI1 LE, as evident in our patient. However, while hyponatremia is commonly found in patients with anti-LGI1 LE, it is not specific [1]. Interestingly, the exact mechanism of hyponatremia is unknown but has been hypothesized to be antibody-mediated due to its reversibility and possibly due to inflammation of the hypothalamic-pituitary neuraxis from direct extension [9].

There are many similar clinical manifestations to anti-LGI1 autoimmune LE, and it is important to be able to distinguish this diagnosis from others such as viral encephalitis and Creutzfeldt-Jakob disease.

With any viral encephalitis, patients typically present with systemic symptoms of infection such as fever as well as headache and behavioral disorders [8]. Previous work has shown that anti-LGI1 LE can initially be clinically indistinguishable from HSV-1 encephalitis. Therefore, in our patient, acyclovir treatment was started empirically, being that HSV encephalitis was in the differential, until CSF findings were negative for HSV-1 and HSV-2.

Lastly, other groups have also shown that sporadic CJD can mimic many features of anti-LGI1 LE, which can further complicate the diagnosis as was the case with our patient. For this reason, a more detailed discussion of CJD will follow.

Specifically, patients with CJD can develop rapidly progressive dementia and cognitive dysfunction with myoclonic movements, Parkinsonism, or ataxia. Patients with CJD may also have hyponatremia (60%). Cerebrospinal fluid with 14-3-3 protein or neuron-specific enolase (NSE) is seen in CJD with high sensitivities, but can also be positive in other paraneoplastic and autoimmune encephalitides [10]. A commonly known EEG pattern of periodic synchronous bi- or triphasic sharp wave complexes (PSWC) is observed in 67 to 95 percent of patients with sporadic CJD (sCJD) at some time during the course of the illness. PSWCs have a very high specificity for the diagnosis of sCJD. Objective diagnostic EEG criteria proposed in 1996 were found to have a sensitivity and specificity of 67 and 86 percent, respectively, for the diagnosis of CJD [11]. Ultimately, a definitive diagnosis is made via a positive prion protein scrapie (PrPSc) staining of brain tissue on histopathology. Currently no treatment is available for CJD making it uniformly fatal if actually the cause of one's symptoms.

Most of these features were present in our patient, so CJD was on the differential at the time of admission, making the diagnosis of anti-LGI1 autoimmune LE in this patient challenging. However, the EEG findings, MRI findings, and disease progression in our case were not consistent with CJD, which is more progressive and fatal. Thus, though CJD was a strong initial differential, it was not compatible with our case.

Many patients with anti-LGI1 LE have normal or minimal CSF findings. If CSF findings are present, one normally sees oligoclonal bands [12].

LGI1 antibody detection in CSF has been found to be lower than that in serum. In cases in which antibody is detected in CSF, its titer is only 1-10% of serum titer, and thus it is crucial that serum antibody tests for autoimmune encephalitis are done to prevent invasive lumbar punctures that may not be needed. Studies have shown that antibody titer levels do not necessarily correlate with disease severity or prognosis [4]. However, though serum antibody positivity may show higher titers, CSF antibody detection has a higher sensitivity (100%) compared to serum antibody (92%) and is thus more useful for diagnostic purposes [13].

Interestingly, studies have shown that 14-3-3 protein can also be found in anti-LGI1 autoimmune LE [10]. One study that examined autopsies of patients suspected to have CJD found that 22 of 384 patients actually had autoimmune encephalitis, and 50% of these encephalitis patients had elevated 14-3-3 protein in the CSF. This shows that 14-3-3 protein is in fact not as specific for diagnosing CJD as once thought and that elevated 14-3-3 protein may be indicative of another disease process such as autoimmune encephalitis [14]. This phenomenon can complicate the diagnosis because prion disease is classically associated with this finding. This dilemma was evident in our case and added complexity to its diagnosis.

MRI findings typically show T2-FLAIR medial temporal lobe findings [15]. About 70% of patients diagnosed with anti-LGI1 LE have increased T2 and FLAIR signals in the temporal lobe or hippocampus, with some extending into the amygdala, striatum, or insula. One study found that approximately 40% of patients also had basal ganglia lesions that correlated to FBDS.

With our patient, her initial MRI was unremarkable, but a repeat one later showed symmetrical signal abnormality with swelling of hippocampi and right mesial temporal lobe, a subtle T2 FLAIR signal abnormality, and low-level restricted diffusion along the insular cortex consistent with temporal lobe pathology.

Eighty percent of EEGs are abnormal including epileptic seizures and slowing. Although there is no definitive EEG pattern specific for anti-LGI1 limbic encephalitis or any other autoimmune encephalitides, the presence of continuous slow waves and frontal intermittent rhythmic delta activity may suggest possible autoimmune etiology for seizures [16]. However, EEG serves as a limited diagnostic tool in distinguishing autoimmune from other causes of seizures and thus clinical manifestations and other diagnostic criteria must be reviewed. In our patient, we did see left temporal focal discharges with focal slowing evolving to electroclinical seizure suggesting left temporal pathology. Follow-up EEG while on treatment showed diffuse generalized slowing and background slowing compatible with generalized cortical dysfunction.

The mainstay of treatment of anti-LGI1 LE generally focuses on steroids, IVIG, and/or plasmapheresis. One series of six patients demonstrated an excellent response to IV methylprednisolone in three out of six patients with significant clinical improvement one week after administration [17]. In another series, all 10 patients who received steroids had good response regardless of treatment with plasma exchange and/or IVIG [18]. The advantage of IVIG treatment and plasmapheresis is that it is unlikely to worsen infectious encephalitis. Kaymakamzade et al. suggest that IV pulse steroids should be tried prior to IVIG or plasma exchange [19], but currently no definitive evidence of superiority has been reported [3].

Other accepted therapeutic options include immunosuppressive agents such as cyclophosphamide and rituximab. Studies have found that rituximab has had favorable outcomes in patients with anti-LGI1 LE, even when administered late in the illness. It is well tolerated in the older adult population and should be heavily considered as a therapeutic option, especially with complications that may arise with the use of IVIG, steroids, and plasma exchange. Additionally, one study also showed favorable outcome with the use of mycophenolate mofetil in decreasing seizure frequency and improving behavioral memory testing [20].

In cases in which concurrent neoplasm is found, treatment with chemotherapy, radiation, and/or surgery can be sought after the underlying autoimmune encephalitis has been managed.

Early detection and aggressive management in patients with anti-LGI1 LE is crucial as appropriate treatment can reverse the disease process and the patient can return to baseline. Studies have found that approximately 80% of patients had improvements in cognitive function and a decrease in seizures after two weeks of receiving first-line therapies such as corticosteroids, IVIG, or plasmapheresis. Recurrence rate was about 30% and usually occurred within the first six months, and mortality rate was 6-19% [4].

In our case, the patient was started on pulse steroids and showed dramatic resolution of symptoms. She showed vast improvement and was discharged after reaching near baseline with minimal cognitive symptoms. The patient was eventually tapered off steroids about a year later, but started showing symptoms of worsening memory and cognitive changes, which was concerning for relapse. At that time, she was given high dose steroids followed by five days of IVIG, which did not show much improvement. Rituximab was considered at this time and rituximab cycles were continued at an interval of six months, and the patient gradually showed improvement and has now returned to baseline.

## Conclusions

This is a unique case presented due to its challenging clinical presentation of abnormal movements and faciobrachial dystonic seizures and conflicting factors such as 14-3-3 positivity. However, a reversible condition, anti-LGI1 LE was diagnosed early, which completely changed the outcome and prognosis for this patient. Our case also showed challenges in management due to the availability of limited studies explaining clear management and treatment options. This case shows that rituximab should be considered as an early treatment option in cases that fail to respond to mainstay treatments such as high dose steroids and IVIG.

## Appendices

Abbreviations

LGI1 - Leucine-rich glioma-inactivated 1 LE - Limbic encephalitis AMS- Altered Mental Status FBDS - Faciobrachial dystonic seizures VGKC - Voltage gated potassium channel EEG - Electroencephalogram ED - Emergency department CBC - Complete blood count GFR - Glomerular filtration rate TSH - Thyroid stimulating hormone RPR - Rapid plasma reagin DOI - Day of Illness HSV - Herpes Simplex Virus RF - Rheumatoid factor ANA - Antinuclear antibody VDRL - Venereal disease research laboratory ANCA - Anti neutrophil cytoplasmic antibody CK - Creatine kinase CMV - Cytomegalovirus EBV - Epstein-Barr virus VZV - Varicella zoster virus CT - Computed tomography MRI - Magnetic Resonance Imaging LP - Lumbar puncture IV - Intravenous IVIG - Intravenous Immunoglobulin FLAIR - Fluid-attenuated inversion recovery WBC - White blood cell AED - Anti-epileptic drug CSF - Cerebrospinal fluid IgG - Immunoglobulin G MVC - Motor vehicle collision
